# When is it time for reverse transcription to start and go?

**DOI:** 10.1186/1742-4690-6-24

**Published:** 2009-03-04

**Authors:** Marylène Mougel, Laurent Houzet, Jean-Luc Darlix

**Affiliations:** 1 Université Montpellier 1, Centre d'études d'agents Pathogènes et Biotechnologies pour la Santé (CPBS), CNRS, UMR 5236, CPBS, 4 Bd Henri IV, CS69033, 34965 Montpellier, France; 2 Molecular Virology Section, Laboratory of Molecular Microbiology National Institute of Allergy and Infectious Diseases, National Institutes of Health, Bethesda, Maryland 20892, USA; 3 LaboRetro, Unité de virologie humaine INSERM U758, IFR128, ENS, 46 allée d'Italie, Lyon, France

## Abstract

Upon cell infection by a retrovirus, the viral DNA polymerase, called reverse transcriptase (RT), copies the genomic RNA to generate the proviral DNA flanked by two long terminal repeats (LTR). A discovery twenty years ago demonstrated that the structural viral nucleocapsid protein (NC) encoded by Gag is an essential cofactor of reverse transcription, chaperoning RT during viral DNA synthesis. However, it is only recently that NC was found to exert a control on the timing of reverse transcription, in a spatio-temporal manner. This brief review summarizes findings on the timing of reverse transcription in wild type HIV-1 and in nucleopcapsid (NC) mutants where virions contain a large amount of newly made viral DNA. This brief review also proposes some explanations of how NC may control late reverse transcription during Gag assembly in virus producer cells.

## Review

### Overview on the process of viral DNA synthesis by RT

**Figure 1:**
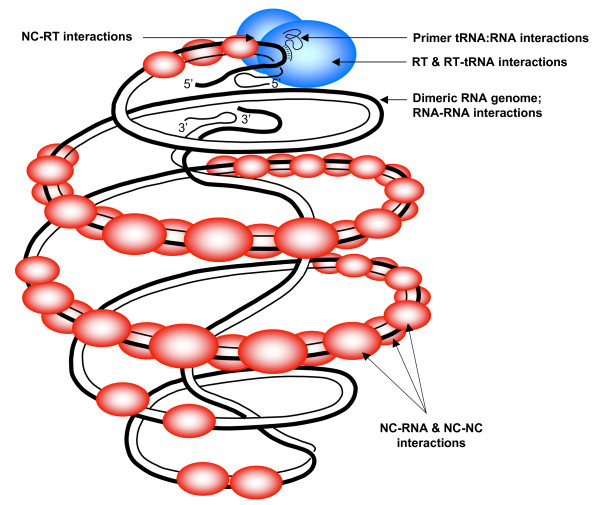
**A scheme of the HIV-1 replication complex**. The genomic RNA in a dimeric form is coated by about 1500 nucleocapsid (NC) protein molecules (in red) in the viral core particle. Molecular interactions have been characterized, at least in part, namely (i) the two viral RNAs via DIS, DLS, 5'-3' and other interactions to form the dimeric 60S complex (black lines), (ii) the primer tRNALys3 (thin black line) annealed to the viral PBS RNA, (iii) NC molecules coating the viral RNA and tRNA (NC basic residues and zinc fingers), (iv) the primer tRNALys3 bound to RTp66-p51(in blue), (v) the viral RNA and RT, (vi) NC and RT and (vii) NC and NC molecules. Viral proteins such as IN and Vif that may play a role in viral DNA synthesis are not represented in this scheme. For references see text.

Retroviruses differ from other positive-strand RNA viruses in the sense that their genomic RNA is reverse transcribed to generate a double stranded DNA flanked by two long terminal repeats (LTR). This essential multistep process is performed by the retroviral RNA/DNA dependent DNA polymerase named reverse transcriptase (RT) discovered in 1970 [1-3]. Originally, the RT activity was found in purified avian and murine virions upon treatment with a low concentration of a non-ionic detergent [1-6]. At almost the same time, RT activity was also found in virus-like particles purified from human fluids and cells [7,8]. Later, an RT-associated RNaseH activity was characterized and found to be relevant to the process of viral DNA synthesis [9,10]. The series of reactions carried out by RT to copy the retroviral genome in order to generate the double stranded viral DNA which is then integrated into the host-cell genome has been known for almost 25 years. Reverse transcription first requires a specific cellular tRNA annealed to the primer binding site (PBS) for the initiation of cDNA synthesis (Figure 1) and two obligatory DNA strand transfers to carry out the synthesis of the complete, LTR flanked, proviral DNA [11-13]. The dimeric nature of the retroviral RNA genome is largely responsible for the high genetic variability of highly replicating viruses such as Rous sarcoma virus (RSV) and HIV-1 by means of forced and non-forced copy-choice recombinations during reverse transcription [12,14,15].

The discovery of HIV-1 and the AIDS epidemic fueled unprecedent interest in and support for basic research on retroviruses as well as extensive efforts aimed at combating the AIDS virus. As a result, the structure-function relationships of HIV-1 RT have been and continue to be intensively studied using a multidisciplinary approach. The 3D structure of RT in its p66-p51 free form was established [15-17], and more recently the specific orientation of the RT polymerase and RNaseH active sites was characterized using single molecule assays *in vitro *[18,19]. Later, it was discovered that the major virion protein of the inner core of alpha and gamma-retroviruses and lentiviruses, the nucleocapsid protein (NC) encoded by Gag was a key cofactor of the RT enzyme, chaperoning obligatory steps in viral DNA synthesis [20-28]. At the same time, the NC domain of the Gag structural polyprotein was found to direct genomic RNA selection, packaging and dimerization during virion assembly [29-34]. Thus NC is a multifunctional virus structural protein necessary for the completion of the early and late phases of retrovirus replication (reviewed in [28,35-38]).

How then can we explain the multiple roles of NC? NC is a potent nucleic acid chaperone, which tightly binds nucleic acids and facilitates the annealing of complementary sequences as well as strand transfer and exchange reactions in physiological conditions (reviewed in [35-38]). NC is encoded by Gag in most, if not all, retroviruses and retrotransposons [36] where its unique chaperoning activity ensures primer tRNA annealing to the genomic PBS and the obligatory minus and plus DNA strand transfers that are required for the synthesis of the complete, LTR flanked, viral DNA [28,37,38].

In the inner core of the HIV-1 viral particle, about 1500 NC molecules [39] coat the genomic RNA in the form of NC oligomers [28,40]. In addition, tight interactions were found to take place between NC molecules, the cellular tRNA primer and the RT enzyme in reconstituted HIV-1 replicative complexes (Figure 1). These multiple RT-NC-RNA interactions contribute to the fidelity of the reverse transcripton reaction by inhibiting self-initiation of cDNA synthesis and providing excision-repair activities to the RT enzyme *in vitro *[24,39,41-46].

After cell infection, the virion core is released into the cytoplasm where it is believed to undergo structural alterations giving rise to a large ribonucleoprotein structure called the reverse transcription complex (RTC), the site of extensive viral DNA synthesis. The RTC is thought to predominantly consist of the genomic RNA coated with NC protein molecules and other components such as the matrix (MA), capsid (CA) and viral protein R (Vpr) molecules together with the viral enzymes RT and integrase (IN) [47-49]. A different view was recently provided by biochemical and electron microscopy studies showing that HIV-1 cores remained in the cytoplasm of newly infected cells up to the nuclear pore [50-54]. These results strongly suggest that completion of proviral DNA synthesis most probably relies on the proper structure and the stability of the viral cores.

### Newly made viral DNA in retroviral particles

The canonical view of retrovirus formation, notably that of HIV-1, states that the overall process takes place at the plasma membrane where Gag molecules assemble via interactions between MA and the phospholipids on the one hand, and between NC and the genomic RNA on the other (reviewed in [55]). Upon completion, immature particles are produced by budding during which Gag and Pol processing by the viral protease (PR) occurs, ultimately leading to the condensation of the inner core [32,56]. However, a series of results indicate that assembly can also take place on intracellular membranes such as endosomes and multivesicular bodies [57-60]. The PR enzyme may therefore already be active at the onset of assembly directing the cleavage of the Gag and Gag-Pol polyproteins, as evidenced by the presence of mature CA, MA and NC proteins in cytoplasmic extracts of infected cells. In both cases, the newly made viral particles are thought to contain the full length viral RNA in a dimeric form as the genetic material along with minor quantities of spliced RNAs [61].

However, small amounts of viral cDNA were found in newly made viral particles of RSV, Moloney murine leukemia virus (MoMLV), and HIV-1 indicating that an active RT enzyme can function during virus assembly [10,62-64]. This notion of premature reverse transcription has been confirmed by Zhang et al. [64] who showed that AZT treatment of HIV-1 infected T cells resulted in a 10–100 fold decrease of the intravirion cDNA level. In addition, the physiological microenvironment, for example the seminal fluid, was found to enhance the accumulation of intravirion viral DNA by a process called natural endogenous reverse transcription (NERT) [64]. Interestingly, synthesis of a full length infectious viral DNA can be achieved in virions of MLV and equine infectious anemia virus (EIAV) under well defined *in vitro *conditions [6,65], that probably reconstitute the microenvironment promoting extensive NERT, especially components present in the seminal fluid [66].

The newly synthesized viral DNA present in infecting virions was shown to play a key role *in vivo *because it augments virus infection of non-activated human primary target cells by nearly one hundred fold while it has no effect on activated T cells [66]. The role of the physiological microenvironment is not limited to viral DNA synthesis since a recently identified aggregating prostatic acidic phosphatase (PAP)-derived peptide that is abundant in the seminal fluid was shown to augment virus to cell attachment and entry, thus facilitating the very early event of HIV-1 infection during a sexual intercourse [67,68].

### HIV-1 NC and the timing of reverse transcription

**Figure 2:**
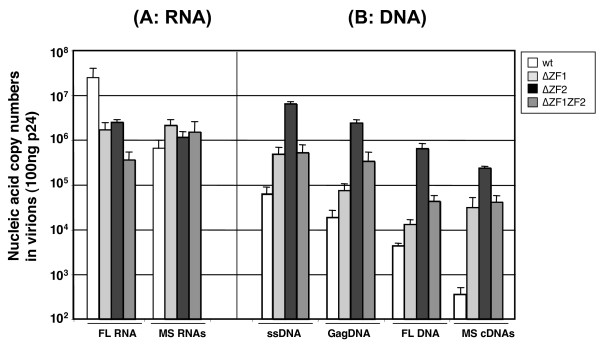
**Nucleic acids content of wild-type and NC mutant HIV-1 virions**. WT, ΔZF1, ΔZF2 and ΔZF1ZF2 represent wild type, and deletion of the first, the second and of both CCHC zinc fingers, respectively. All values were determined by quantitative RT-PCR (A) and PCR (B) (n = 3 ± SD). A- Viral RNAs corresponding to the full length (FL) and the multispliced (MS) forms. B- Viral DNAs corresponding to the strong stop (ss), Gag, full length (FL) and mutispliced (MS) forms.

It has long been shown that mutating the highly conserved CCHC residues of the NC zinc fingers impairs genomic RNA packaging and results in the production of replication defective viral particles (reviewed in [28,37,38]). Moreover, mutations affecting the 3D structure of the zinc fingers or their respective orientation cause a decrease in the genomic RNA content of HIV-1 viral particles and result in the production of defective particles, although significant amounts of viral DNA can be synthesized in infected cells ([26,27,45,69]; reviewed in [37,38,70]). However, this has been recently disputed. In fact, the influence of NC mutations on unsuspected aspects of HIV-1 virion formation has just been discovered, in particular the influence on packaging of multispliced 1.8 kb RNA (MS RNA) and NERT [71,72]. It was found that deleting or mutating the NC zinc fingers or the N-terminal basic residues caused a 10–30 fold reduction of genomic RNA in newly made virions (Fig. 2A), while this had no or a slightly positive influence on virion incorporation of MS RNA that contain part of the packaging Psi element recognized by NC (Fig. 2A). At the same time there was a 10–100 fold enhancement of newly synthesized viral DNA in these NC mutant HIV-1 virions as compared with wild type virions (Fig. 2B; [72,73]). This unexpected accumulation of viral DNA in NC mutant virions was independently reported by R. Gorelick and colleagues [74].

This reverse transcription takes place in virus producer cells since the addition of the RT inhibitor AZT prevented accumulation of viral DNA in virions, in agreement with the earlier findings of Pomerantz et al. [66] on wild type HIV-1 virions. A closer examination of the viral DNA made in these NC mutant virions reveals that large viral DNA fragments accumulated (Fig. 2B) together with cDNA generated by reverse transcription of the incorporated multispliced viral RNAs. A fraction of this viral DNA was found to be functional since it directed Tat synthesis and LTR activation in human cells [72].

These findings not only confirm the key role of NC in RT-directed viral DNA synthesis and probably its maintenance ([43,45]; reviewed in [70]) but also indicate that NC exerts a control on the timing of reverse transcription. How then can we explain this extensive reverse transcription already in cells producing the HIV-1 NC mutant virions? Within HIV-1 virions with mutations in or deletion of the NC CCHC zinc fingers, the core is formed of mature Gag proteins but it is mostly globular and does not adopt a condensed cone-shaped structure as seen by electron microscopy [75]. These results favor the notion that these NC mutations cause a defect in the late step of Gag assembly. The fact that NC ZF mutants have lost, at least in part, their high binding affinity for the viral RNA [76] and also for the LTR DNA ends [77] could explain the condensation defect of the inner core structure and degradation of the LTR DNA ends [45]. Such ZF mutations also have a negative impact on NC-RT interactions *in vitro *[42,77] while the chaperoning activities of the NC mutant proteins either are not or are slightly affected ([78]; reviewed in [37]). The study of Gorelick et al. [74] also showed that mutating the PTAP motif in the Gag C-terminal p6 domain caused a budding delay probably due to a loosening of the interactions between Gag and the cellular transporter protein TSG101 and at the same time led to a strong activation of premature reverse transcription.

**Figure 3:**
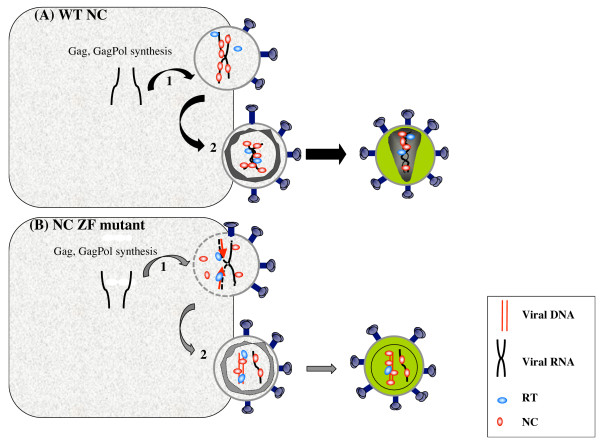
**A proposed mechanism for late reverse transcription in cells producing HIV-1 NC mutant particles**. A- Newly made Gag and GagPol molecules (1) assemble using the genomic RNA and cellular membrane as platforms, (2) then wild type HIV-1 virions are produced by budding. These processes are facilitated by interactions between NC and cellular proteins (large black arrows). The core containing the genomic RNA is condensed with a cone-shaped structure. A limited level of viral DNA synthesis in producer cells and also anatural endogenous reverse transcription (NERT) can occur (see text). B- In cells producing HIV-1 Gag and GagPol with mutation in or deletion of the nucleocapsid CCHC zinc finger (1), assembly (2) and budding (grey arrow) are probably slowed down due to impaired interactions between NC and the viral RNA. They result in a partial delocalization of Gag in producer cells and a reduced level of newly made viral particles (grey arrow) (see text for references). The resulting virion core is formed of mature Gag proteins, but it is poorly condensed as seen by electron microscopy (see text for references). Taken together, these observations favor the notion that the NC CCHC mutations modify the kinetics of viral assembly which prevent core condensation and could explain, at least in part, why late-premature reverse transcription can readily take place in producer cells.

Taken together, these findings support the notion that the highly conserved CCHC zinc fingers of NC control formation of a dense core structure where reverse transcription is prevented, at least, partially [72-74]. Thus, the viral NC protein would exert a control on the timing of viral DNA synthesis by the active RT enzyme, delaying the start phase in virus producer cells and chaperoning the entire process until completion in newly infected cells (Figure 3). There are also indications that the conserved CCHC zinc fingers of NC contribute to the maintenance of the complete viral DNA and its IN-mediated integration into the host genome during the course of cell infection [45,79,80].

### Future prospects

One outstanding avenue of research is to better understand, at the molecular level, the multiple interactions between NC, RT, and the viral nucleic acids that ensure fidelity and completion of viral DNA synthesis (reviewed in [70]). It is noteworthy that these molecular interactions (Fig. 1) are probably involved in recombinations, by forced and unforced DNA strand transfers via the NC-chaperoning activity, that fuel the genetic diversity of the progeny virus [12,14]. The zinc fingers and the RT palm domains are probably required for NC-RT recognition [42-45,81]; yet, the exact interacting domains remain to be determined *in vitro *and in the viral context.

The physiological microenvironment appears to greatly facilitate HIV-1 NERT. In fact, seminal plasma was found to activate NERT, virus-cell attachment, and entry [64,66-68]. The presence of molecules such as dNTP, polyamines and a prostatic acidic phosphatase-derived peptide called SEVI in seminal fluid would be responsible, at least in part, for the strong enhancement of HIV-1 infectivity on non-activated primary target cells (op.cit). Yet, the molecular and biochemical mechanisms of such an enhancement in virus infectivity remain to be determined.

A further important avenue of research involves the characterization of compounds aimed at inhibiting HIV-1 RT-NC interactions, and thus the chaperoning [82] of reverse transcription in virus producer cells and in the reverse transcription complex (RTC) in newly infected cells [83]. This work is presently ongoing.

Loosening the interactions between NC protein molecules, the genomic RNA and the RT enzyme by mutating the NC zinc fingers activates viral DNA synthesis in HIV-1 producer cells and results in the production of DNA-containing particles [72-74], similar in nature to foamy viruses (HFV, SFV) where viral DNA synthesis occurs in virus producer cells [84-86]. This finding raises several questions on the nature of functional RT-NC-RNA interactions in most, if not all, retroviruses, notably alpha- and gammaretroviruses such as ASLV and MLV's that contain a mature NC protein. For Foamy viruses, the situation appears much different since RT would have to interact with Gag since it is not itself processed as it is in NC-containing retroviruses. Moreover, no NC-like chaperoning activity has yet been characterized. The structure of HFV/SFV Gag resembles that of Gag of the fruit fly retrovirus Gypsy where the C-terminal domain has NC-like RNA chaperoning activities, but lacks a zinc finger [87]. Thus it would be interesting to examine the putative interaction, if any, between the C-terminal NC-like domain of Gypsy and Foamy virus Gag with the homologous RT enzyme.

Endogenous retroviruses and retrotransposons form a large fraction (42%) of the mammalian genome, but only a very small fraction (<1%) of these endogenous retroviral sequences is expressed and capable of producing viral particles that can be infectious [88,89]. Nonetheless, the continuous expression of these retroviral sequences in their natural form [90,91] or as reconstructed, highly active recombinants [92,93] could be detrimental to the host, possibly causing a variety of diseases [94-97]. Thus endogenous retroviruses such as Gypsy, MLV and HERV which can undergo retrotransposition at a low frequency and behave similarly to exogenous retroviruses would represent interesting model systems to study the spatio-temporal control of viral DNA synthesis by cellular factors [98-100].

## Competing interests

The authors declare that they have no competing interests.

## Authors' contributions

MM, LH and JLD equally participated in writing the intellectual content and drafting the manuscript. All authors read and approved the manuscript in its final form.
